# Mechanism and early intervention research on ALI during emergence surgery of Stanford type-A AAD: Study protocol for a prospective, double-blind, clinical trial

**DOI:** 10.1097/MD.0000000000005164

**Published:** 2016-10-21

**Authors:** Yi Cheng, Mu Jin, Xiuhua Dong, Lizhong Sun, Jing Liu, Rong Wang, Yanwei Yang, Peirong Lin, Siyu Hou, Yuehua Ma, Yuefeng Wang, Xudong Pan, Jiakai Lu, Weiping Cheng

**Affiliations:** a Department of Anesthesiology; b Department of Cardiology; c Department of Epidemiology, Beijing Anzhen Hospital, Capital Medical University, Beijing Institute of Heart Lung and Blood Vessel Diseases, Beijing, China.

**Keywords:** acute lung injury, coagulation/fibrinolysis, inflammatory, Stanford type-A acute aortic dissection

## Abstract

**Background:**

Stanford type-A acute aortic dissection (AAD) is a severe cardiovascular disease demonstrating the characteristics of acute onset and rapid development, with high morbidity and mortality. The available evidence shows that preoperative acute lung injury (ALI) induced by Stanford type-A AAD is a frequent and important cause for a number of untoward consequences. However, there is no study assessing the incidence of preoperative ALI and its independent determinants before Standford type-A AAD surgery in Chinese adult patients.

**Methods/design:**

This is a prospective, double-blind, signal-center clinical trial. We will recruit 130 adult patients undergoing Stanford type-A AAD surgery. The incidence of preoperative ALI will be evaluated. Perioperative clinical baselines and serum variables including coagulation, fibrinolysis, inflammatory, reactive oxygen species, and endothelial cell function will be assayed. The independent factors affecting the occurrence of preoperative ALI will be identified by multiple logistic regression analysis.

**Trial registration:**

ClinicalTrials.gov (https://register.clinicaltrials.gov/), Registration number NCT01894334.

## 1 Background

Patients presenting with aortic dissection within 2 weeks of the onset of symptoms are considered to have acute aortic dissection (AAD).^[1]^ AAD is a severe cardiovascular disease demonstrating the characteristics of acute onset and rapid development, with high morbidity and mortality. It has been shown that mortality of patients with AAD per hour early after the onset of symptoms is about 1% to 2%. Furthermore, mortality of patients with AAD who do not receive surgery is 58.0% in contrast to 26% in surgically treated patients.^[2,3]^ In patients with AAD, multiple organ failure including acute lung injury (ALI) is frequent.^[4,5]^ It is reported that 51% of patients meet the criterion of oxygenation impairment (oxygen fraction [PaO_2_/FiO_2_] ≤ 200 mm Hg) within 24 hour after onset of distal type AAD.^[6]^ Actually, preoperative ALI has been shown as a frequent complication associated with a number of untoward consequences, such as prolonged ventilator support, longer stay in intensive care unit and hospital, and even induces death. The previous work by Wang et al^[7]^ showed that the incidence of postoperative hypoxemia was up to 49.5% in Chinese adult patients undergoing Stanford type-A AAD surgery and postoperative hypoxemia was attributable to preoperative disturbance in oxygenation (78.5%, PaO_2_/FiO_2_ ≤ 200 mm Hg). In addition, preoperative hypoxemia can also result in surgery delay, which increases the risk of aortic rupture, cardiac tamponade, or even death. Therefore, the prophylaxis and/or treatment of preoperative ALI induced by AAD is important for final outcomes of patients.

Better understanding of the pathophysiology of preoperative ALI in the patients with AAD can improve clinical prophylaxis and/or treatment, but its underlying molecular and cellular mechanisms remain largely unknown. It is reported that the occurrence of oxygenation impairment on hospital days 2.5 in patients with Standford type-B AAD can be predicted by a multivariate logistic regression model.^[8]^ To our best knowledge, however, there is no study having assessed whether the preoperative ALI in Chinese adult patients undergoing the Standford type-A AAD surgery can be predicted by preoperative clinical and basic assessments. Thus, we put forward a prospective study strategy aiming to address the above issues.

## 2 Aims

The aims of this study are to clarify the incidence, independent risk factors, and possible mechanisms of preoperative ALI in Chinese adult patients undergoing the Standford type-A AAD surgery. This will include a detailed statistical analysis of perioperative clinical baselines and serum variables, such as coagulation, fibrinolysis, inflammatory, reactive oxygen species, and endothelial cell function. The outcomes of this study will obtain the details regarding the incidence, independent predictors, and pathophysiological mechanisms of preoperative ALI, which will benefit the prophylaxis and/or treatment of preoperative ALI induced by Standford type-A AAD.

## 3 Methods

### 3.1 Design of trial

**Figure 1:**
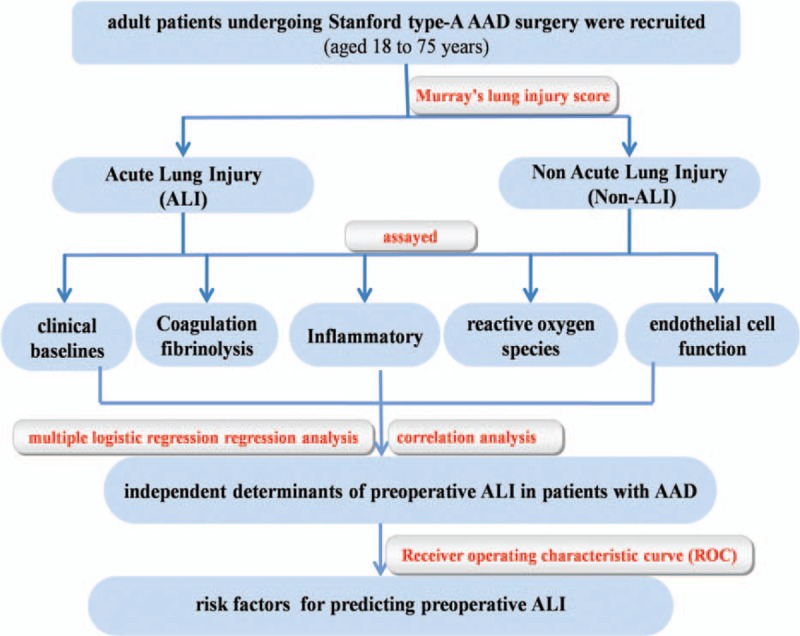
The flowchart of the trial.

This trial is a prospective, double-blind, signal-center clinical trial which had been registered at www.clinicaltrials.gov (identifier NCT01894334) and approved by the Beijing Anzhen Hospital Clinical Research Ethics Committee (Fig. 1).

### 3.2 Participants (inclusion and exclusion criteria)

The sample of subjects is estimated to 130 patients, based on a 79% incidence of ALI before surgery in patients with Stanford type-A AAD^[7]^ and the set power to 80% for a type I error of 5% (Power Analysis and Sample Size, PASS V11.0, NCSS, LLC. Kaysville, UT). A convenient sample will be recruited from the Beijing Anzhen Hospital. The AAD surgery in the Beijing Anzhen Hospital is performed in approximately 300 cases per year. If 30% of these patients are eligible and consent, we will meet our recruitment target in about 1.5 years, which can avoid the bias from the different levels of surgical procedures in different periods.

Patients aged 18 to 75 years will be included. The patients with coronary heart disease; heart failure; severe cardiac tamponade; unstable hemodynamics; nervous system abnormalities; clinically apparent malperfusion^[9]^ including lower limb, cerebral, coronary, and renal malperfusion; and visceral ischemia will be excluded. All patients enrolled in this study have normal hepatic and renal function according to the preoperative physical and laboratory examinations. No patient is prescribed with nonsteroidal anti-inflammatory drugs or corticosteroids before or after admission. All patients will be informed and consented with regard to their participation in the study.

### 3.3 Anesthetic managements

To avoid aortic rupture, systolic blood pressure will be controlled between 100 and 120 mm Hg with oral β-blockers and intravenous infusion of vasodilators (nitroprusside, etc.) before surgery. On surgery day, morphine hydrochloride 10 mg will be injected intramuscularly 1 hour before entering operation room. After entering the operating room, patients will be monitored with a 5-lead electrocardiography, pulse oximetry (SpO_2_), bispectral index (BIS), and invasive blood pressure through a left radial artery catheter. After tracheal intubation, a right internal jugular central venous catheter will be placed for monitoring of central venous pressure.

Anesthesia will be induced with intravenous midazolam, etomidate, sufentanil, and neuromuscular blockade drugs. After tracheal intubation, general anesthesia will be maintained with intravenous sufentanil, propofol, and either isoflurane or sevoflurane. BIS will be maintained at between 45 and 55 throughout surgery. The patient’ lungs will be mechanically ventilated with a FiO_2_ 100% oxygen and a 5-cmH_2_O positive end-expiratory pressure (PEEP). The end-tidal carbon dioxide pressure will be maintained at between 35 and 45 mm Hg by adjusting tidal volume of 6 to 8 mL/kg and respiratory rate of 10 to 15 times/min. During the cardiopulmonary bypass (CPB), mechanical ventilation will be discontinued and pulmonary static inflation (5 cmH_2_O) will be performed with compressed air.

### 3.4 Surgical procedure^[10,11]^

Patients undergo median sternotomy and CPB. Cannulation of the femoral artery and/or right axillary artery will be performed for CPB. Furthermore, the right axillary artery cannulation will also be carried out for selective cerebral perfusion. After CPB is established and cooling is initiated, the ascending aorta will be clamped and cardiac arrest will be accomplished with cold cardioplegic solution during the cooling phase. When the nasopharyngeal temperature is decreased to 20 °C to 25 °C, circulatory arrest will be instituted. A stent graft and a 4-branch prosthetic graft will be used for arch replacement. After the open distal anastomosis is completed, the 4-branch prosthetic graft will be cross-clamped. Then the blood perfusion of the lower body will be started via the perfusion limb of the 4-branch prosthetic graft. Selective cerebral perfusion will be continued until the anastomosis to the left common carotid artery, innominate artery, and left subclavian artery is accomplished. Then CPB will be resumed to normal flow, and rewarming will be started. Valve-sparing root resection (aortic valve plasty, the David procedure, or valsalva sinus plasty) or valve graft replacement will be performed according to the subtype of the aortic root.

### 3.5 Definitions

In all patients, the diagnosis of AAD will be confirmed by history, chest radiography, transthoracic, and contrast-enhanced computed tomogrphy or magnetic resonance imaging. The dissection is considered as AAD if the time from the onset of the symptoms to surgery is 14 days or less. This study will only include the type-A AAD according to the Stanford classifications.^[1,2]^

The definition criteria of the ALI includes an acute hypoxemic respiratory insufficiency with bilateral pulmonary infiltrates, but is not caused by left atrial hypertension according to the left ventricular ejection fraction (LVEF). The ALI will be defined using Murray lung injury score (Murray LIS).^[12,13]^ Murray LIS consists of 4 distinct clinical parameters, including the bilateral infiltrates observed on chest radiograph, oxygenation index (PaO_2_/FiO_2_ ratio), lung compliance, and PEEP value. The severity of ALI was quantified as follows: no = the Murray LIS 0, mild to moderate = the Murray LIS 0.1 to 2.5, and acute respiratory distress syndrome = the Murray LIS > 2.5. The Murray LIS will be calculated using a specially designed tool on the web site (http://med.39.net/tools/murray.html).

Hypertension is defined as systolic blood pressure ≥140 mm Hg and/or diastolic blood pressure ≥90 mm Hg, or the need of antihypertensive agents.^[14]^ Diabetes mellitus is defined as fasting glucose level ≥126 mg/dL (7.0 mmol/L), or a glycosylated hemoglobin (Hb) ≥6.5%,^[15]^ or treatment with oral hypoglycemic agents or insulin. Smoking status will be defined as current smokers according to self-report. A glomerular filtration rate less than 60 mL/min/1.73 m^2^, and increase in serum creatinine by more than 50% times baseline is defined as impaired renal function.^[16]^

## 4 Data collection

Participants are reviewed at face-to-face assessments at recruitment. If a participant is unable to attend a face-to-face assessment, a person with legal responsibility will be a substitute. A secondary review of all ALI cases and a random sample of non-ALI cases will be performed by an independent expert investigator who is blinded to the initial ALI adjudication. The following data will be collected through the assessments:

### 4.1 Demographics and etiology

Height, weight, the body mass index (BMI), regular use of prescription drugs (include antihypertensive agents, hypoglycemic agents or insulin, anticoagulant, and nonsteroidal anti-inflammatory drugs), smoking history (age commenced, age ceased, and average cigarettes smoked per day), hypertension history (age of onset and treatment), and diabetes history (age of onset and treatment) will be recorded at recruitment.

### 4.2 Preoperative assessment

#### 4.2.1 Time from onset of symptoms to surgery

The onset time of sudden severe chest or upper back pain (often described as a tearing, ripping, or shearing sensation that radiates to the neck or down the back), loss of consciousness, shortness of breath, sudden difficulty speaking, loss of vision, weakness or paralysis of 1 side of the body will be recorded.

#### 4.2.2 Hemodynamics

The invasive blood pressure will be recorded as a baseline immediately after the left radial artery catheter.

#### 4.2.3 Echocardiogram

The data of preoperative echocardiogram will be recorded, including aortic diameter, LVEF, and left ventricular end-diastolic dimension (LVEDd).

#### 4.2.4 Operative risk assessment

In this prospective study, each participant will be assessed with the European system for cardiac operative risk evaluation (EuroSCORE).

#### 4.2.5 Murray lung injury score assessment

After routinely anesthesia and tracheal intubation, based on a FiO_2_ of 100%, a PEEP of 5 cmH_2_O, a tidal volume of 8 mL/kg, a 1:2 of inspiratory/expiratory ratio, and a respiratory rate of 10 per minute, the Murray LIS is obtained by radiographic findings on admission, oxygenation index (a PaO_2_/fraction of FiO_2_ ratio), lung compliance, and PEEP value.^[12,13]^

### 4.3 Surgical data

The durations of surgery, CPB and aortic cross-clamping, and lowest temperature during CPB will be recorded. The blood transfusion, intravenous colloid, and crystalloid volumes and uine volume during surgery will also be noted.

### 4.4 Postoperative data

During a 24-hour postoperative period, the blood transfusion, intravenous colloid, and crystalloid volumes and uine volume will be calculated. The duration of postoperative mechanical ventilation and in-hospital mortality will also be recorded.

### 4.5 Serum measurements

A 10-mL venous blood sample will be drawn from central vein catheter immediately after tracheal intubation. After rapidly centrifugation, plasma will be obtained and stored immediately at −70 °C condition for further analysis. Plasma levels of interleukin-6, interleukin-10, tissue factor, tissue factor pathway inhibitor, plasminogen activator inhibitor type 1, human leukocyte elastases, tumor necrosis factor α, vascular endothelial growth factor, prostaglandin I2, and thromboxane B2 will be assayed by enzyme-linked immune absorbent assay according to the manufacturer's specification (MULTISKAN MK3 Automatic microplate reader, Thermo Fisher Scientific Inc, Waltham, MA). Methane dicarboxylic aldehyde, myeloperoxidase, total antioxidation capacity, and total superoxide dismutase will be determined by ultraviolet spectrophotometry. The platelet count, Hb, white blood cells, glucosamine, lactic acid, troponin, fibrinogen (FIB), FIB degradation product, d-dimer, active part thrombin time, and prothrombin time will be determined by standard quantitative assay techniques in the hospital Clinical Laboratory Center.

## 5 Statistical analysis

The data obtained in this study will be analyzed using SPSS 18.0 (SPSS Inc., Chicago, IL). Univariate and multiple logistic regression analyses will be used to determine the independent predictors of preoperative ALI. Univariate analysis will be applied for demographic data (age, sex, and BMI), comorbidities (smoking history, hypertension, and diabetes), preoperative data (hemodynamics, onset of symptoms to surgery, aortic diameter, LVEF, LVEDd, and EuroSCORE), surgical data (surgery time, hypothermic circulatory arrest time, intravenous colloid, crystalloid, and transfusion volumes), postoperative variables (intravenous colloid, crystalloid and transfusion volumes, urine volume, seroma volume of drainage, mechanical ventilation time, and in-hospital mortality), and serum measurements before surgery (coagulation/fibrinolysis, inflammatory, ROS, platelet, and endothelial cell function). In univariate analysis, variables associated with preoperative ALI with a *P* value <0.10 will be selected. Then, these selected variables will be put into the multiple logistic regression analysis to identify the independent predictors of preoperative ALI.

Receiver operating characteristic curve will be used to test discrimination of multivariate regression model and to determine the cutoff value in predicting preoperative ALI with sensitivity and specificity, respectively. The missing values will be displaced by means according to Intention-To-Treat rules. A *P* value <0.05 will be considered statistically significant.

## 6 Discussion

Population-based studies suggest an incidence of AAD of about 3 cases per 100,000 people per year in the United States of America.^[1]^ According to this incidence, there are almost 400,000 cases per year in China, which indicates over 20,000 patients with preoperative ALI per year. However, there has been no reliable incidence of preoperative ALI of such patients in China. Our study will be conducted in one of the largest clinic center of cardiovascular diseases in China, and a large nationally representative sample of the Standford type-A AAD in China will be collected. Therefore, the outcome of this trial can present the clinical characteristics of preoperative ALI in Chinese adult patients undergoing the Standford type-A AAD surgery.

In patients with AAD, ALI is a usual complication associated with untoward consequences or even leads to losing the chance of surgery. Although the risk factors and mechanisms of ALI are still unclear, the available evidence shows that systemic inflammatory response is activated through AAD surgery.^[4,17]^ Inflammation is a protective response essential for maintaining human health and fighting disease, but excessive inflammatory response can promote the irreversible cellular damage. The extensive activation of leckocyte and uncontrolled release of proinflammatory cytokines can increase the permeability of the vessel wall and thrombosis, which will contribute to the organs damage. Since the pulmonary vascular bed is an important reservoir of neutrophils, the lungs tend to be a major site of tissue damage by AAD.

Recent works have shown that coagulation/fibrinolysis and inflammation are host defense mechanisms, mutually dependent in mounting an adequate immune response against potentially injurious challenges.^[18]^ Furthermore, secondary fibrinolysis can aggravate ALI.^[19,20]^ Thus, we hypothesize that both the inflammation and coagulation are involved in ALI by AAD. By the statistical adjustment and analysis, it can be anticipated that findings of this study may reveal the related mechanisms of ALI by AAD. If these hypotheses are confirmed in our further clinical study, the treatments aimed to the pathophysiology of preoperative ALI can be recommended to decrease perioperative morbidity and mortality by ALI in patients with AAD. Thus, our findings have potentially clinical implications.
